# Editorial: *Vibrio* Species in the Food Processing Chain

**DOI:** 10.3389/fmicb.2021.796796

**Published:** 2021-11-25

**Authors:** Antonio Valero, Francisco N. Arroyo-López, Marta López Cabo, Sheng Chen, Ilenys M. Pérez-Díaz

**Affiliations:** ^1^Department of Food Science and Technology, University of Cordoba, Córdoba, Spain; ^2^Food Biotechnology Department, Instituto de la Grasa (CSIC), Seville, Spain; ^3^Laboratory of Microbiology and Technology of Marine Products (MICROTEC), Instituto de Investigaciones Marinas (IIM-CSIC), Vigo, Spain; ^4^Department of Infectious Diseases and Public Health, City University of Hong Kong, Hong Kong, Hong Kong SAR, China; ^5^USDA-ARS-SEA-Food Science and Market Quality and Handling Research Unit, Raleigh, NC, United States

**Keywords:** *Vibrio* spp., antibiotic resistance, sequencing techniques, virulence genes, foodborne illness

Rising concern about the foodborne illnesses caused by pathogenic *Vibrio* species (mainly *V. parahaemolyticus, V. cholera*, and *V. vulnificus*) has led to a strengthening of research on the characterization of the presence of the genus in food matrices, virulence genes, pandemic markers, and the correlation between clinical and environmental isolates from different ecosystems. The emergence of antimicrobial resistance strains (AMR) in *Vibrio* spp. may produce a decrease in the effectiveness of commonly used antibiotics, thus posing a threat to public health. Progress in genomic studies has identified motile elements implied in gene transfer that may give birth to developing surveillance strategies for risk mitigation. The development of new infection models that can predict the pathogenesis of *Vibrio* spp. and the use of high-throughput sequencing techniques for serogroup genes may be useful tools for understanding molecular pathways and the infectivity of *Vibrio* spp. food isolates. In this Research Topic, different approaches, aiming at characterizing *Vibrio* spp. from aquaculture, marine, and vegetable ecosystems, together with the evaluation of microbial behavior and the development of new infection and serogroup models, are shown.

A mini-review by Dutta et al. discusses the role and antimicrobial resistance of pathogenic *Vibrio* spp. They present potential sources of antibiotic resistance genes for *Vibrio* spp., including the horizontal gene transmission from other pathogens as the main route. This has shown the genetic basis of the emergence of multidrug and extensively multidrug resistant *Vibrio* spp. through different types of highly mobile elements that can be extensively propagated among bacteria. The use of phage or probiotic therapies as alternative treatments for the inactivation of antibiotic resistant species of *Vibrio* may be helped by the maintenance of good hygiene practices and processing technologies to protect public health.

Antibiotic resistance genes can also originate from the environment, such as wastewater effluents or sediments in marine or aquaculture habitats. In this regard, Siddique et al. studied the characterization of pathogenic *V. parahaemolyticus* in a fish farm ecosystem (tilapia, rui, and shrimp). Among the 216 samples, 60.2% were positive for the pathogen, including 323 isolates of which 17 harboured the *trh* virulence gene gene. They confirm the presence of resistant strains to amoxicillin, ampicillin, and penicillin. Pathogenicity was further confirmed by the fluid accumulation in the ileal loop of rabbits being O8: KUT, the most predominant pathogenic serotype.

The presence and characterization of *V. parahaemolyticus* and *V. vulnificus* in marine and estuarine environments was studied by da Silva et al. They found 150 isolates of *V. parahaemolyticus*, including 52 positives for *trh* gene, and 129 of *V. vulnificus* from water and blue crab samples. PFGE and agglutination tests were used for molecular subtyping and determination of antibiotic resistance. The study showed the high presence of the O5 pathogenic serotype, together with the multidrug resistant isolates (41%) and the high genetic diversity of both *Vibrio* species, as no correlations were found among the sampling sites, antimicrobial resistance profiles, and pathogenicity.

The associated presence of *Vibrio* spp. in water ecosystems may underestimate their origin from other environmental and food sources. Ready-To-Eat vegetables can harbor pathogenic *Vibrio* spp. if poor manufacturing, hygiene, and storage practices are followed. Igbinosa et al. evaluated the presence of *V. parahaemolyticus* in minimally processed vegetables. Among the 63 isolates, they found microbial counts from 1.5 to 1,000 MPN/g and drug resistant isolates to ampicillin and cefotaxime mainly (>60%). They studied the biofilm formation finding that 23.8% of the isolates were strong biofilm producers. Regarding the presence of virulence genes, 100, 14.3, and 31.8% of the isolates harbored the *toxR* gene, *trh*, and *tdh* determinants, respectively.

The microbial behavior of *Vibrio* spp. can be quantified with predictive models. Posada-Izquierdo et al. investigated the fate of a *Vibrio* spp. cocktail inoculated in lye-treated table olives for 22 days. A predictive growth model was developed as a function of salt concentration (2–12%) and pH (4–9) using a synthetic medium and table olive brines. They found a higher effect of salt concentration than of pH for the growth inhibition of *Vibrio* spp. However, they were not able to proliferate in the table olives during fermentation, highlighting that phenolics compounds could exert a clear antimicrobial effect.

The disposal of reliable models to predict the pathogenesis of *Vibrio* spp. are increasingly needed since the use of virulence markers could not fully elucidate the presence of long-standing virulence indicators. This was demonstrated by Santos et al. using clinical and environmental *V. parahaemolyticus* isolates in two systemic infection models, namely mice and *Galleria mellonella* larvae. Interestingly, non-pathogenic environmental isolates produced lethal infections regardless of their source, serotype, and genotype (*tdh, orf8, toxRSnew*, and *vpadF*). A high correlation was found in the assayed models, supporting that *G. mellonella* larvae can be used as an alternative model to study the pathogenesis of *V. parahaemolyticus*.

Recently, the use of high-throughput sequencing technologies has aided researchers in deciphering the genome of different species. This was essential to provide complete knowledge of the molecular and metabolic pathways of microorganisms and the identification of virulence gene clusters. Bian et al. have developed VPsero, a rapid serotyping tool for *V. parahaemolyticus* using serogroup specific genes obtained from whole-genome sequencing data. The algorithm, based on the comparison of lipopolysaccharide and capsular polysaccharide gene clusters covered 43 K and 12 O serogroups. The authors showed the high sensitivity and specificity of the tool (>0.91), though limitations could be faced in future studies, such as the addition of new serogroups, the verification of the quality of assembled genomes and the availability of short reads.

This Research Topic presents a collection of manuscripts highlighting relevant findings in the pathogenesis of *Vibrio* spp. in the food chain and suggests future directions for research, enabling progress in the development of novel analytical methods and surveillance actions to mitigate the emerging risk posed by these human pathogens.

## Author Contributions

All authors listed have made a substantial, direct, and intellectual contribution to the work and approved it for publication.

## Conflict of Interest

The authors declare that the research was conducted in the absence of any commercial or financial relationships that could be construed as a potential conflict of interest.

## Publisher's Note

All claims expressed in this article are solely those of the authors and do not necessarily represent those of their affiliated organizations, or those of the publisher, the editors and the reviewers. Any product that may be evaluated in this article, or claim that may be made by its manufacturer, is not guaranteed or endorsed by the publisher.

